# Effective glucose metabolism maintains low intracellular glucose in airway epithelial cells after exposure to hyperglycemia

**DOI:** 10.1152/ajpcell.00193.2019

**Published:** 2019-08-21

**Authors:** Jade Bearham, James P. Garnett, Victoria Schroeder, Matthew G. S. Biggart, Deborah L. Baines

**Affiliations:** ^1^Institute for Infection and Immunity, St. George’s University of London, London, United Kingdom; ^2^Institute of Cellular Medicine, Newcastle University, Newcastle upon Tyne, United Kingdom; ^3^Immunology and Respiratory Diseases Research, Boehringer Ingelheim Pharma and Company, Biberach an der Riss, Germany

**Keywords:** airway, epithelial cell, glucose, hexokinase, metabolism

## Abstract

The airway epithelium maintains differential glucose concentrations between the airway surface liquid (ASL, ~0.4 mM) and the blood/interstitium (5–6 mM), which is important for defense against infection. Glucose primarily moves from the blood to the ASL via paracellular movement, down its concentration gradient, across the tight junctions. However, there is evidence that glucose can move transcellularly across epithelial cells. Using a Förster resonance energy transfer sensor for glucose, we investigated intracellular glucose concentrations in airway epithelial cells and the role of hexokinases in regulating intracellular glucose concentrations in normoglycemic and hyperglycemic conditions. Our findings indicated that in airway epithelial cells (H441 or primary human bronchial epithelial cells) exposed to 5 mM glucose (normoglycemia), intracellular glucose concentration is in the micromolar range. Inhibition of facilitative glucose transporters (GLUTs) with cytochalasin B reduced intracellular glucose concentration. When cells were exposed to 15 mM glucose (hyperglycemia), intracellular glucose concentration was reduced. Airway cells expressed hexokinases I, II, and III. Inhibition with 3-bromopyruvate decreased hexokinase activity by 25% and elevated intracellular glucose concentration, but levels remained in the micromolar range. Exposure to hyperglycemia increased glycolysis, glycogen, and sorbitol. Thus, glucose enters the airway cell via GLUTs and is then rapidly processed by hexokinase-dependent and hexokinase-independent metabolic pathways to maintain low intracellular glucose concentrations. We propose that this prevents transcellular transport and aids the removal of glucose from the ASL and that the main route of entry for glucose into the ASL is via the paracellular pathway.

## INTRODUCTION

Glucose concentrations in the airway surface liquid (ASL) of a healthy individual are typically 0.4 mM, 12.5 times lower than plasma glucose concentrations (5 mM), but this has been shown to rise during periods of hyperglycemia and inflammation (1, 31). Previous studies have shown that the appearance of glucose in the ASL is largely reliant on paracellular movement of glucose via tight junctions, down its concentration gradient (19, 30). However, there is some evidence that glucose can also move transcellularly across the airway epithelium from the blood to the ASL via glucose transporters in the cellular membrane (19, 22, 30). Such a process is found in other systems such as the intestine and the kidney (where glucose moves from the lumen to the blood) although the gradient driving transcellular movement of glucose in these tissues is in the opposing direction to that of the lung (16, 27). We hypothesized that transcellular movement of glucose in the airway is largely dependent on the intracellular concentration of glucose, which is regulated by hexokinase activity. Low intracellular glucose maintains a driving force for glucose to enter the cell. However, if intracellular glucose concentrations rise to that of ASL or higher, for example, during exposure to hyperglycemia, this would promote luminal efflux of glucose. Understanding the routes for glucose movement across the airway epithelium is vital because an increase of glucose in the ASL has been associated with increased airway infections in respiratory disease (3, 5).

Glucose Förster resonance energy transfer (FRET) sensors have been developed to exhibit a change in fluorescence output upon glucose binding, indicating a change in local glucose concentrations. These sensors have been used to measure intracellular glucose concentrations in systems such as ovarian epithelial cells (4) and glucose fluxes in pancreatic β-cells (21). To our knowledge, intracellular glucose concentrations in airway epithelial cells and the metabolic processes regulating intracellular glucose concentrations have not yet been investigated.

In this study, we used a FRET sensor to measure intracellular glucose concentrations in airway epithelial cells in normoglycemic and hyperglycemic conditions. We also investigated the involvement of hexokinases in regulating intracellular glucose concentration, airway cell glucose metabolism, and the effect on ASL glucose concentrations.

## MATERIALS AND METHODS

### 

#### Cell culture.

H441 airway epithelial cells were cultured at 37°C, 5% CO_2_, in RPMI 1640 media containing 10 mM glucose and supplemented with 10% fetal calf serum (Sigma-Aldrich), 2 mM l-glutamine, 1 mM sodium pyruvate, 5 µg/mL insulin, 2.75 µg/mL penicillin, and 100 mg/mL streptomycin (Life Technologies). Human bronchial epithelial cells (HBECs) were originally purchased from Lonza and Epithelix before semi-immortalization with polycomb complex protein BMI-1 (BMI-1) transduction and were cultured in collagen-coated flasks (Corning) in bronchial epithelial growth media (BEGM; Lonza) in a humidified environment at 37°C, 5% CO_2_. Growth media was replaced every second day, and cells were passaged once 80% confluent.

Polarized monolayers were cultured on Transwells (Corning). H441 cells were plated onto the Transwell using the medium described above until confluent. The apical medium was then removed, and the basolateral medium was changed to RPMI 1640 media containing 10 mM glucose and supplemented with 4% charcoal stripped serum, 200 µM dexamethasone, 10 nM 3,3′-5-triiodothyronine, 2 mM l-glutamine, 1 mM sodium pyruvate, 5 µg/mL insulin, 2.75 µg/mL penicillin, and 100 mg/mL streptomycin. Cells were then cultured at air-liquid interface (ALI) for 10 days, changing the medium every other day until the cells formed a resistive monolayer. HBECs were seeded at a density of 200,000 cells/cm^2^ on Transwells. After confluence was achieved, media were removed from the apical surface, and the cells were fed on the basolateral side only with 50% BEGM and 50% high-glucose minimal essential medium containing 100 nM retinoic acid. The media were exchanged every 2–3 days, and the apical surface mucus was removed by gentle washing with phosphate-buffered saline once a week. Cultures were used for functional analysis 28–35 days after exposure to ALI. BMI-1-transduced cells exhibit normal cell morphology, karyotype, and doubling times despite extensive passaging. When cultured at ALI, they show normal ciliation, show normal production of MUC5AC and MUC5B, and have electrophysiological properties similar to primary cells (26). Transepithelial resistance was measured before use with the epithelial volt/ohmmeter EVOM (Word Precision Instruments), and at least 200 Ω·cm^2^ resistance was required before use in experiments. Eighteen hours before experiments, cell media were exchanged with growth medium containing 5 mM d-glucose (supplemented as listed above). To investigate the effect of hyperglycemia, cells were either exposed to 5 mM d-glucose + 10 mM l-glucose (an analog not transported or metabolized, to control for any osmotic effects of raising glucose) to mimic normoglycemia (5 mM glucose) or 15 mM d-glucose to mimic hyperglycemia (15 mM glucose). The apical surface of cell cultures was gently washed with 100 μL PBS to obtain airway surface liquid washes. Glucose in the washes was analyzed using an Amplex Red glucose oxidase kit (Thermo Fisher).

#### Cell transfection.

Cells were seeded at a density of 2 × 10^5^ cells/cm^2^ onto glass coverslips coated in polylysine and, once at 50–65% confluency, were transiently transfected with 1 µg of the glucose-sensitive sensor FLII12Pglu-700µΔ6 (Addgene plasmid no. 17866) or cyan fluorescent protein-yellow fluorescent protein (CFP-YFP) FRET positive control plasmid (a kind gift from R. Tarran, University of North Carolina at Chapel Hill, Chapel Hill, NC) using Lipofectamine 2000 (Thermo Fisher). Polarized monolayers were apically transfected in a similar fashion, with 1 µg of plasmid transfected using TransIT-X2 (Mirus) applied to the apical surface of the cells.

#### FRET microscopy.

Cells were imaged 48–72 h posttransfection in phosphate-buffered saline at 37°C, 95% air-5% CO_2_, supplemented with glucose and/or inhibitors using a Zeiss LSM 510 Meta confocal microscope with a ×20 Plan-Neofluar lens or a Leica SP8 with a ×20 PL APO CS2 lens. FLII12Pglu-700µΔ6 contains the FRET paired fluorophores enhanced CFP (eCFP; donor) and Citrine (acceptor), which report a reduced eCFP-to-Citrine FRET ratio with a binding of glucose. This was measured on the Zeiss LSM 510 by collecting emission data from eCFP (459–505 nm) and Citrine (525–600 nm) every 4 s over an 8-min time period while exciting eCFP at 458 nm. Settings were optimized for the growth conditions of each cell type, which took into account opacity of the substrate (i.e., glass coverslips and Transwells), cell height, and cell density. Thus, the output measurement was different for the three conditions studied.

#### Generating dose-response data for the sensor.

Glucose dose-response data were generated for each cell type and growth condition. Cells transfected with FLII12Pglu-700µΔ6 were treated with hexokinase inhibitor 3-bromopyruvic acid (BrPy, 100 µM) plus the respiratory chain complex I inhibitor rotenone (100 nM) for 30 min to inhibit glucose metabolism. During this time, cells were incubated with different glucose concentrations to equilibrate intracellular glucose with extracellular glucose before imaging as previously described to equilibrate intracellular and extracellular lactate for FRET measurement (33). FRET activity of FLII12Pglu-700µΔ6 was imaged as described above.

#### Hexokinase assay.

Cells were untreated or pretreated for 10 min with BrPy (0.1 µM to 1 mM) at 37°C, 95% air-5% CO_2_. Cell lysates were prepared, and a colorimetric hexokinase assay (ab-136957; Abcam), which measures the conversion of glucose to glucose-6-phosphate by hexokinase, was performed as per the manufacturer’s instructions.

#### Sorbitol assay.

Proliferating H441 cells were exposed to 5 mM d-glucose + 10 mM l-glucose or 15 mM d-glucose in the presence or absence of BrPy (100 µM) for 10 min before washing in ice-cold PBS. Cells were then lysed in 200 µL of assay buffer and centrifuged for 5 min at 4°C at 12,000 rpm. The lysate was decanted, and sorbitol concentrations were determined by sorbitol colorimetric assay (ab-118968; Abcam) as per the manufacturer’s protocol.

#### Seahorse glycolysis stress assay.

Human bronchiolar epithelial cells were seeded into a Seahorse XF96 plate and incubated at 37°C, 5% CO_2_ for 48 h. The medium was changed 24 h before the Seahorse experiment, and cells were exposed to 5 or 15 mM glucose with or without BrPy (100 μM) or epalrestat (1 or 10 μM) for the last 30 min before the Seahorse glycolysis stress assay was performed according to the manufacturer’s instructions followed by the sequential injection of oligomycin to inhibit ATP-linked reparation and 2-deoxy-d-glucose (2-DG) to inhibit glucose metabolism. The plate layout was separated into quadrants to reduce edge effects. Extracellular acidification rate (ECAR) and oxygen consumption rate (OCR) were measured. Glycolysis rate was calculated by subtracting the normalized ECAR values after 2-DG injection from the ECAR values after glucose injection to exclude the nonglycolytic acidification from the calculation. Glycolytic capacity was calculated by subtracting the nonglycolytic acidification rate (ECAR after 2-DG injection) from the maximum ECAR after 1 μM oligomycin injection.

#### Western blots.

Cells were lysed in RIPA buffer [20 mM Tris·HCl (pH 7.5), 150 mM NaCl, 1 mM Na_2_EDTA, 1 mM EGTA, 1% Nonidet P-40, and 1% sodium deoxycholate] plus protease inhibitor cocktail (Sigma) with gentle agitation at 4°C for 30 min. Protein concentration was calculated from a bicinchoninic acid assay (Thermo Fisher). Twenty micrograms of protein were electrophoresed through a 4–12% Bis-Tris gel. Gels were blotted onto a PVDF membrane and blocked with Odyssey blocking buffer (Li-Cor). Membranes were incubated in primary antibodies [hexokinase I (HKI), ab-65069, 1:500; hexokinase II (HKII), ab-37593, 1:250; hexokinase III (HKIII), ab-126217, 1:500; and β-actin, A-5441, 1:10,000] followed by secondary antibodies (goat anti-rabbit 680RD, 925-68071, 1:15,000; and donkey anti-mouse 800CW, 925-32212, 1:15,000). Blots were imaged using the Li-Cor Odyssey system.

#### Data analysis.

FRET eCFP/Citrine intensity and Western blot band intensity data were measured using ImageJ software. Data are displayed as means ± standard deviation and analyzed using GraphPad Prism 7 using ANOVA followed by a post hoc Tukey’s test unless otherwise stated.

## RESULTS

### 

#### Hexokinase proteins I, II, and III are present in airway epithelial cells.

**Fig. 1.**
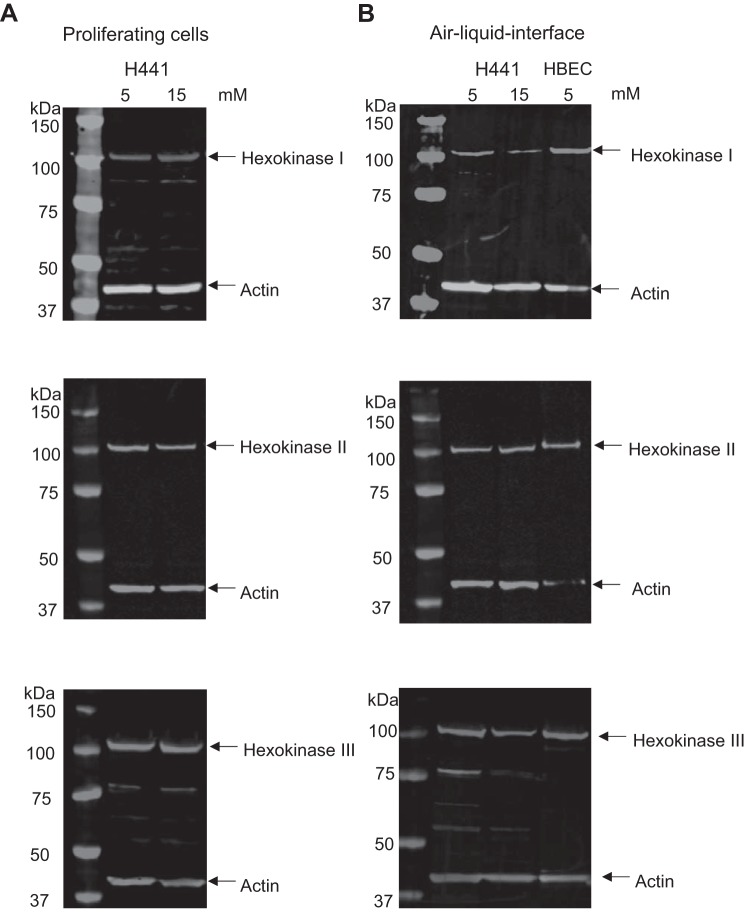
H441 cells and human bronchial epithelial cells (HBECs) express all three forms of hexokinase. Representative Western blots of cell lysates from H441 cells grown on plastic (*A*) or H441 cells or HBECs grown at air-liquid interface (*B*). Lanes indicate cell type and growth conditions of either 5 or 15 mM glucose as indicated. Proteins immunostained for hexokinases I, II, and III are indicated to the right of the blots (all ~100 kDa). The immunostained housekeeping protein β-actin is also indicated (Actin) and serves as a loading control.

As glucose enters the cell, it is phosphorylated by hexokinases to glucose-6-phosphate reducing the intracellular concentration of free glucose. Western blot of cell extracts from H441 cells grown on plastic (proliferating) or H441 cells and HBECs grown at air-liquid interface indicated the presence of hexokinases I, II, and III in these cells. There was no observed difference in the total cellular abundance (hexokinase/actin) of these proteins in H441 cells after exposure to either 5 or 15 mM glucose (Fig. 1, *A* and *B*).

#### Hexokinase activity in airway cells is reduced by BrPy.

**Fig. 2.**
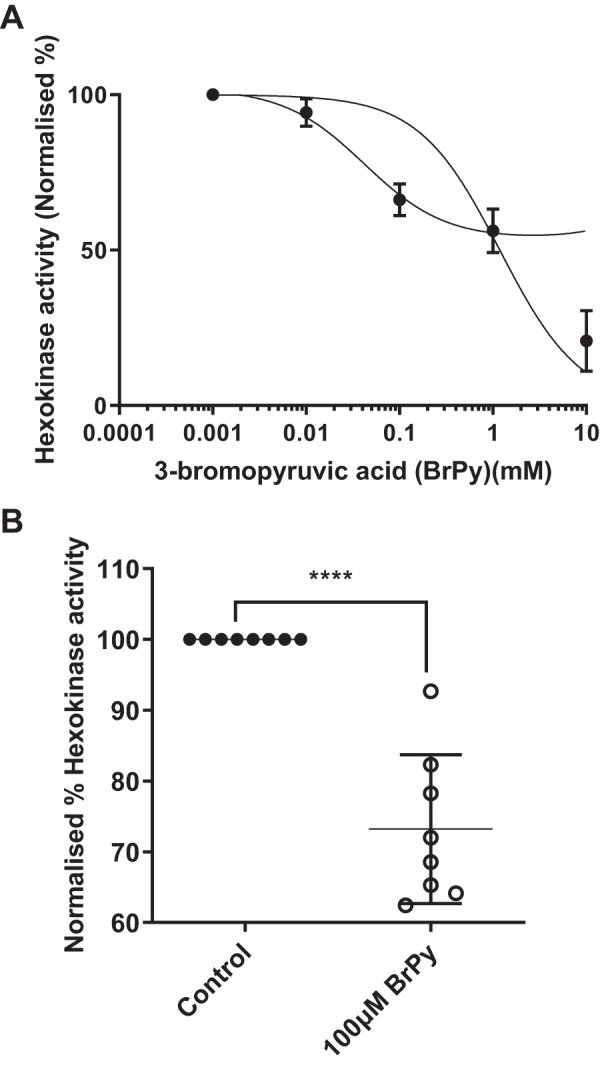
Hexokinase activity is inhibited by 3-bromopyruvic acid (BrPy). *A*: effect of BrPy concentration on hexokinase activity in cell extracts from H441 cells exposed to 5 mM glucose. The dose-response data did not follow a classic sigmoid curve, and there was an indication that the inhibition was biphasic. Two curves could be fitted to the data to reflect initial inhibition (left-hand curve) with an IC_50_ of 0.04 ± 0.01 mM or overall inhibition (right-hand side) with an IC_50_ of 1.2 ± 0.28 mM (*n* = 4 experiments). *B*: total hexokinase activity in cell extracts from control (●) or BrPy (100 μM)-treated cells (○). Individual data points are shown with mean ± SD. ****Significantly different from control, *P* < 0.0001.

Addition of BrPy to H441 cells reduced total hexokinase activity in cell extracts in a dose-dependent manner with an IC_50_ of 1.2 ± 0.28 mM (Fig. 2*A*). The data did not follow a classic sigmoid curve, and there was an indication that the inhibition was biphasic. We were unable to unambiguously fit such a curve to the data. However, the IC_50_ obtained from the initial inhibition of hexokinase activity was lower at 0.04 ± 0.01 mM. As there was no statistical difference in hexokinase activity between pretreatment with 100 µM and pretreatment with 1 mM, it was decided to use the lower concentration of BrPy. At this concentration, total cellular hexokinase activity was reduced by 25.1 ± 11.6% in H441 cells cultured at air-liquid interface (*n* = 6 experiments; Fig. 2*B*).

#### Hexokinase activity drives glycolysis in airway cells.

**Fig. 3.**
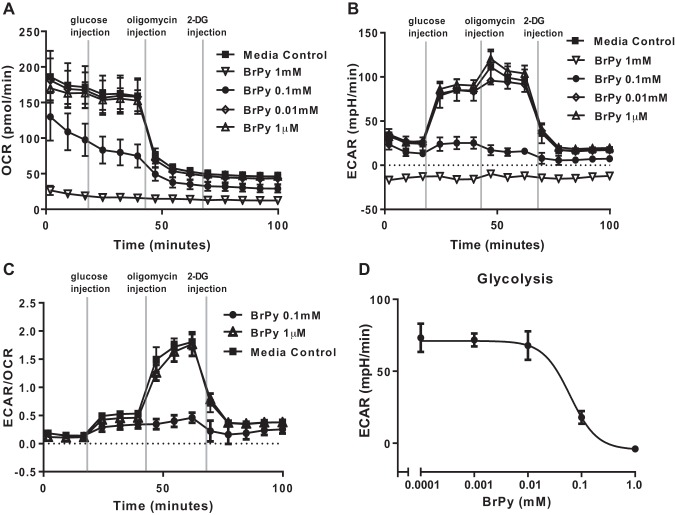
3-Bromopyruvic acid (BrPy) inhibits glycolysis in airway epithelial cells. Seahorse metabolic assay of airway cells exposed to medium or different concentrations of BrPy (1 μM to 1 mM) as indicated on the right-hand side of graphs. *A*–*C*: oxygen consumption rate (OCR, *A*), extracellular acidification rate (ECAR, *B*), and ECAR/OCR (*C*) before and after injection of 5 mM glucose, oligomycin, or 2-deoxy-d-glucose (2-DG) at points indicated. *D*: dose-response data of glycolysis to BrPy were fit with a sigmoidal curve (df = 25, *r*^2^ = 0.95) with an IC_50_ of 0.06 ± 0.02 mM. All *n* = 5 experiments.

Using the Seahorse assay, we previously showed that airway cells produce energy by mitochondrial respiration (OCR) and that elevation of extracellular glucose shifts metabolism to glycolysis (ECAR), which is associated with increased lactic acid secretion (12). We found that BrPy (100 μM) was effective at inhibiting both mitochondrial respiration (Fig. 3*A*) and glycolysis in these cells (Fig. 3, *B* and *C*). We calculated that BrPy inhibited glycolysis with an IC_50_ of 0.06 ± 0.02 mM (Fig. 3*D*). Application of 2-DG, an inhibitor of all hexokinase activity, was more effective at inhibiting respiration and glycolysis (Fig. 3, *A–C*). These data indicate that glycolysis is predominantly driven by hexokinase II activity in these cells.

#### Elevating extracellular glucose and inhibiting hexokinase activity changed FRET ratio in nonpolarized and polarized H441 cells and HBECs.

**Fig. 4.**
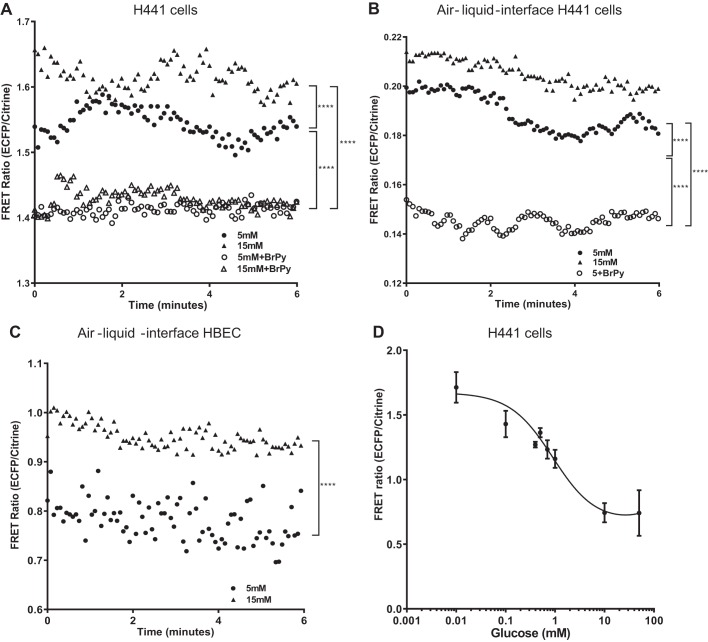
Förster resonance energy transfer (FRET) ratio [enhanced cyan fluorescent protein (eCFP)/citrine] was measured over a period of 6 min using the glucose FRET sensor FLII12Pglu-700µΔ6. *A*: H441 cells grown on coverslips were exposed to either osmotically balanced 5 mM glucose (●) or 15 mM glucose (▲), both *n* = 16 experiments. Cells were also exposed to the same conditions in the presence of the hexokinase inhibitor 3-bromopyruvic acid (BrPy; ○ or △, respectively), both *n* = 14 experiments. *B*: FRET ratio (eCFP/Citrine) for H441 cells grown at air-liquid interface and exposed to either 5 mM glucose (●, *n* = 12 experiments) or 15 mM glucose (▲, *n* = 6 experiments). Cells were also exposed to 5 mM glucose in the presence of the hexokinase inhibitor BrPy (○, *n* = 4 experiments). *C*: FRET ratio (eCFP/Citrine) in human bronchial epithelial cells (HBECs) grown at air-liquid interface, exposed to either osmotically balanced 5 mM glucose (●, *n* = 12 experiments) or 15 mM glucose (▲, *n* = 15 experiments). *D*: FRET ratio (eCFP/Citrine)-glucose dose-response curve for cells shown in *A*, equilibrated with extracellular glucose as described in results. Data points are shown as means only in *A*, *B*, and *C* for clarity. Data in *D* are shown as means ± SD. Data were fitted with a sigmoidal one-site binding curve (df = 37, *r*^2^ = 0.6). Values shown in *A* and *B* are directly comparable, but FRET ratio values in *A*, *B*, and *C* cannot be directly compared because of the different imaging conditions required for the two cell types and their growth substrates. ****Significantly different between groups as indicated, *P* < 0.0001.

Proliferating H441 cells transfected with FLII12Pglu-700µΔ6 and exposed to 5 mM extracellular glucose exhibited a cyclic fluctuation in FRET ratio of eCFP/Citrine over time, with a full cycle taking 3.4 ± 0.2 min (*n* = 16 experiments; Fig. 4*A*). This was not observed when the control FRET eCFP/Citrine plasmid was transfected into cells (data not shown). Elevation of extracellular glucose to 15 mM resulted in an increase in FRET ratio from 1.54 ± 0.02 to 1.6 ± 0.02 (*P* < 0.0001, *n* = 117 individual cells from *n* = 16 experiments), indicating a decrease in intracellular glucose. In addition, the cyclic fluctuations slowed to 4.3 ± 0.3 min for a full cycle (*n* = 16 experiments; *P* < 0.05; Fig. 4*A*). Pretreatment with the hexokinase inhibitor BrPy decreased FRET from 1.54 ± 0.02 to 1.41 ± 0.01 (*P* < 0.0001; *n* = 117 individual cells from *n* = 14 experiments) indicating that intracellular glucose was increased (Fig. 4*A*). Furthermore, BrPy prevented the large cyclic fluctuations in FRET indicating that hexokinase activity was associated with this phenomenon. As an alkylating agent, it is possible that BrPy could directly affect the sensor. However, this would likely reduce glucose binding or stoichiometric changes to the sensor, neither of which would explain these results. Thus, these data indicate that intracellular glucose concentration fluctuated with external glucose concentration and hexokinase activity.

H441 cells cultured at air-liquid interface on permeable supports required altered microscope conditions for FRET acquisition, which meant that the measured FRET ratio of eCFP/Citrine was decreased compared with that observed in proliferating cells. Nevertheless, in cells exposed to 5 mM extracellular glucose the pattern of response was similar to that seen in proliferating cells. A cyclic fluctuation in FRET ratio was also observed in these cells with a full cycle taking 4.4 ± 0.6 min, in 5 mM glucose. Elevation of extracellular glucose to 15 mM resulted in an increased FRET ratio from 0.38 ± 0.007 to 0.41 ± 0.005 (*P* < 0.0001, *n* = 83 individual cells from *n* = 6 experiments). Addition of BrPy reduced FRET ratio to 0.34 ± 0.003 and the cycling frequency to 1.3 ± 0.23 min (*P* ≤ 0.001; *n* = 16 experiments).

Optimization of FRET acquisition in HBECs cultured at air-liquid interface also resulted in a change in FRET ratios obtained. However, similar to H441 cells, FRET ratio increased when extracellular glucose was increased from 5 to 15 mM (*P* < 0.0001, *n* = 149 individual cells).

#### Inhibition of glucose transporter-mediated glucose uptake increased FRET ratio in H441 cells grown at air-liquid interface.

**Fig. 5.**
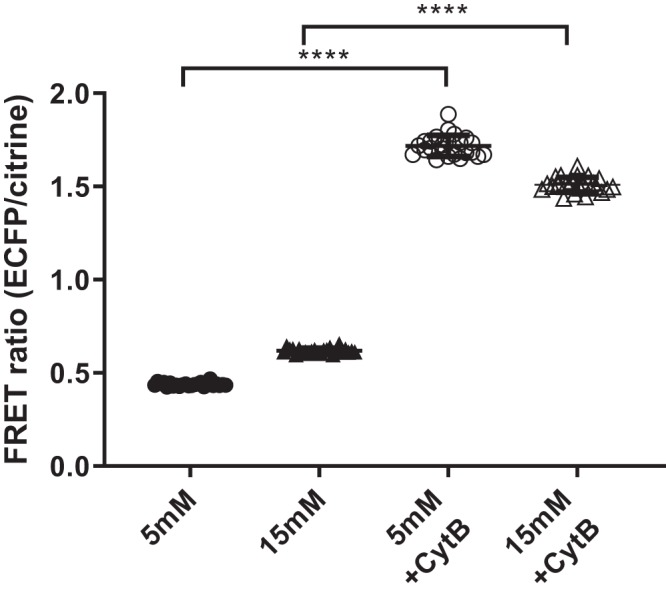
Inhibition of cellular glucose uptake increased Förster resonance energy transfer (FRET) ratio [enhanced cyan fluorescent protein (eCFP)/Citrine] indicating a decrease in intracellular glucose concentration. H441 cells grown at air-liquid interface and exposed to either 5 mM glucose or 15 mM glucose in the absence or presence of the facilitative glucose transport inhibitor cytochalasin B (CytB). Individual data points are shown with mean ± SD; *n* = 24 individual cells. ****Significantly different between groups as indicated, *P* < 0.0001.

Cytochalacin B is a molecule larger than glucose, which binds to the pore of facilitative glucose transporters (GLUTs) and blocks glucose uptake. Cytochalacin B treatment of H441 cells grown at air-liquid interface and exposed to 5 or 15 mM glucose significantly increased FRET ratio (*P* < 0.0001, *n* = 24 individual cells, respectively). These data indicate that inhibition of glucose uptake into the cell reduced intracellular glucose (Fig. 5).

#### Intracellular glucose concentration of H441 cells and HBECs.

**Fig. 6.**
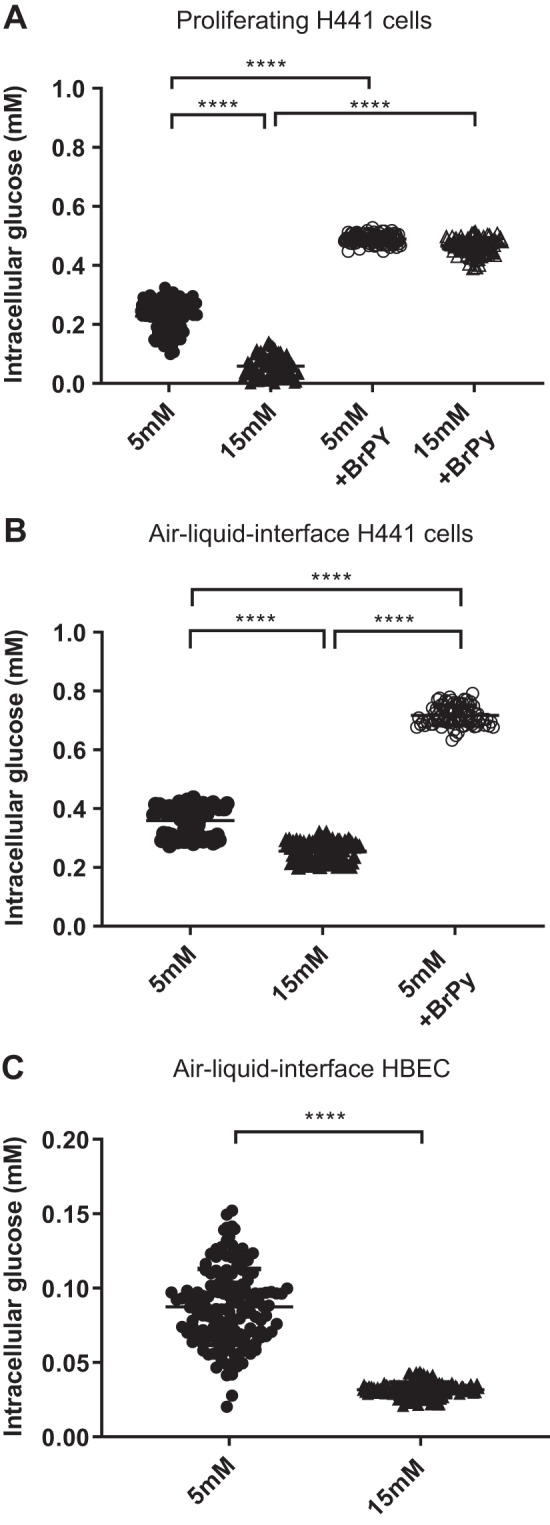
Intracellular glucose concentration calculated from Förster resonance energy transfer ratio dose-response curves. *A*: calculated intracellular glucose concentration in H441 cells grown on plastic and exposed to 5 mM (●) or 15 mM d-glucose (15 mM; hyperglycemia, ▲) or exposed to the same conditions in the presence of 3-bromopyruvic acid (BrPy; ○ or △, respectively). Values were calculated using the dose-response curve shown in Fig. 4*D*. Individual data points are shown with mean ± SD; *n* = 117 individual cells. ****P < 0.0001 between groups as indicated. *B*: calculated intracellular glucose concentration for H441 cells grown at air-liquid interface in either 5 mM glucose (●), 15 mM glucose (▲), or 5 mM glucose in the presence of BrPy (○). Individual data points are shown with mean ± SD; *n* = 83 individual cells. ****P < 0.0001 between groups as indicated. *C*: calculated intracellular glucose for human bronchial epithelial cells (HBECs) cultured at air-liquid interface in either 5 mM glucose (●) or 15 mM glucose (▲). Individual data points are shown with mean ± SD; *n* = 150 individual cells. ****P < 0.0001 between groups as indicated.

A dose-response curve for FRET ratio was generated for the three different cell/growth conditions using the individual imaging conditions used. An exemplar dose-response curve for proliferating H441 cells is shown (Fig. 4*B*). This was then used to interpolate the data points shown in Fig. 4*A* to calculate the intracellular concentration of glucose. The mean intracellular glucose concentration for proliferating H441 cells in 5 mM glucose was 0.23 ± 0.05 mM. Raising the glucose concentration to 15 mM glucose resulted in a decrease in intracellular glucose to 0.05 ± 0.04 mM. Pretreatment with BrPy increased intracellular glucose concentration to 0.49 ± 0.01 mM in 5 mM glucose and 0.46 ± 0.03 in 15 mM glucose (*P* < 0.0001 compared with control, respectively; *n* = 117 individual cells; Fig. 6*A*).

Interpolation of data from H441 cells cultured at air-liquid interface indicated that these cells had a mean intracellular glucose concentration of 0.36 ± 0.005 mM in 5 mM basolateral glucose and this decreased to 0.26 ± 0.003 mM when basolateral glucose concentration was increased to 15 mM. Addition of BrPy in the presence of 5 mM basolateral glucose increased intracellular glucose concentration to 0.72 ± 0.003 mM (*P* ≤ 0.0001; *n* = 83 individual cells; Fig. 6*B*).

A similar pattern was seen in HBECs grown at air-liquid interface. Intracellular glucose concentration was 0.09 ± 0.002 mM in 5 mM glucose, and this decreased to 0.03 ± 0.001 mM when basolateral glucose concentration was raised to 15 mM (*n* = 150 individual cells; Fig. 6*C*).

#### Glucose metabolism.

**Fig. 7.**
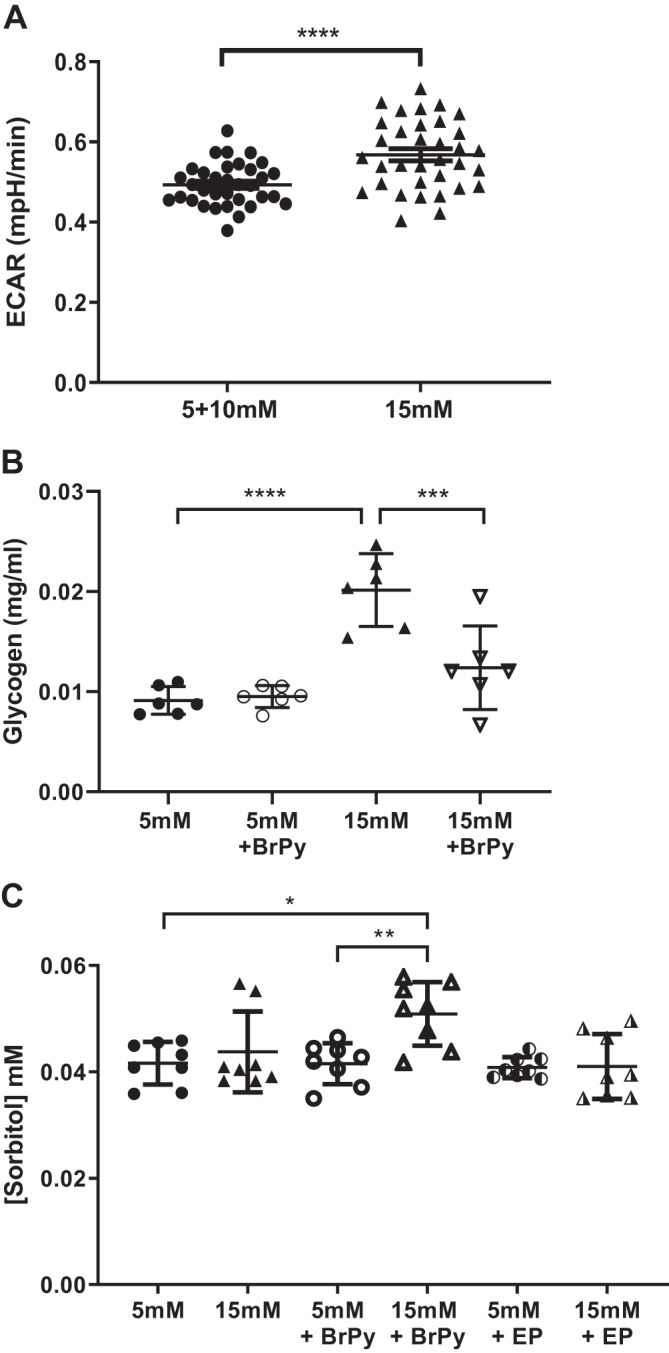
Glycolysis, glycogen, and sorbitol are increased by elevation of extracellular glucose concentration. *A*: glycolysis measured in airway cells as extracellular acidification rate (ECAR) after injection of 5 mM glucose (●) or 15 mM glucose (▲); *n* = 34 experiments. ****P < 0.0001. *B*: glycogen measured in airway cells after exposure to 5 mM glucose (●) or 15 mM glucose (▲) and 3-bromopyruvic acid (BrPy; ○ or ▽, respectively). Individual data points are shown with mean ± SD; *n* = 6 experiments. ***P < 0.001, ****P < 0.0001. *C*: sorbitol measured in airway cells after exposure to 5 mM glucose (●) or 15 mM glucose (▲) and BrPy (○ or △, respectively) or epalrestat (EP; half-shaded symbols). Individual data points are shown with mean ± SD; *n* = 8 experiments. *P < 0.05, **P < 0.01.

Glycolysis was increased in HBECs in response to elevation of extracellular glucose concentration from 5 to 15 mM consistent with our previous observations in H441 cells (Fig. 7*A*; 12). In addition, the amount of glycogen per culture was increased twofold after exposure to 15 mM glucose (from 9.1 ± 1.3 to 20.2 ± 1.5 mg/mL, *P* < 0.0001, *n* = 6 experiments). Inhibition of hexokinase with BrPy (100 µM) reduced glycogen in H441 cells exposed to 15 mM (*P* < 0.001, *n* = 6 experiments) but not 5 mM glucose (Fig. 7*B*). Thus, elevation of extracellular glucose increased hexokinase-driven glycolysis and glycogen synthesis.

Hexokinase-independent pathways are also present in airway cells, such as the polyol pathway, which utilizes aldose reductase to convert glucose to sorbitol. Such a pathway could also contribute to maintaining low intracellular glucose in the face of increased extracellular glucose. There was no significant difference in mean intracellular sorbitol between cells grown in 5 or 15 mM glucose. However, inhibition of hexokinase activity with BrPy in the presence of 15 mM glucose caused a small but significant elevation of sorbitol (from 0.04 ± 0.001 to 0.05 ± 0.002, *P* < 0.01, *n* = 8 experiments). This elevation was inhibited by the aldose reductase inhibitor epalrestat (30 μM; *n* = 8 experiments; Fig. 7*C*). These data indicate that under circumstances when intracellular glucose rises, the sorbitol pathway can contribute to glucose utilization in these cells.

#### Airway surface liquid glucose.

**Fig. 8.**
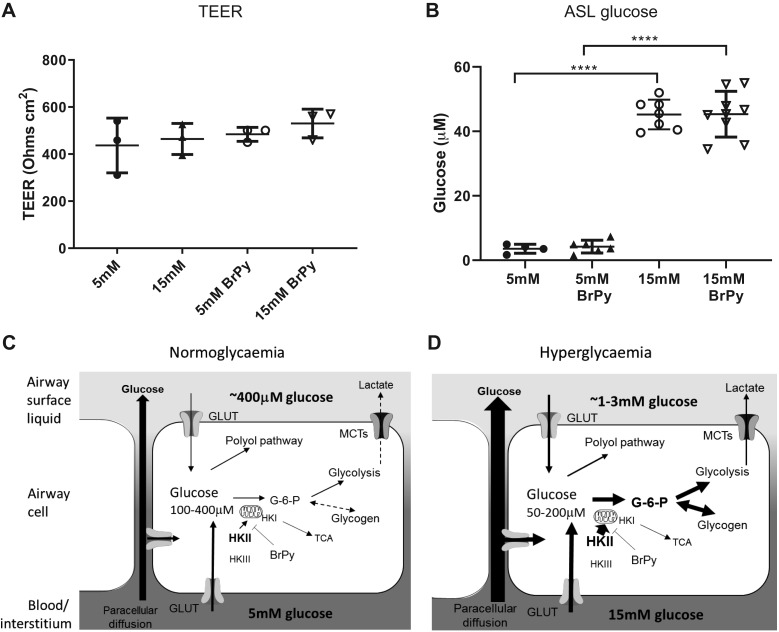
Paracellular diffusion drives airway surface liquid (ASL) glucose concentration. *A* and *B*: transepithelial electrical resistance (TEER, *A*) and glucose concentration (*B*) in ASL washes after exposure to 5 mM glucose (●) or 15 mM glucose (▲) and 3-bromopyruvic acid (BrPy; ○ or ▽, respectively). Individual data points are shown with mean ± SD; *n* = 6 experiments. ****P < 0.0001. *C* and *D*: proposed mechanism for the role of hexokinase II (HKII) in maintaining low intracellular glucose in normoglycemia (*C*) and hyperglycemia (*D*). There is a diffusion gradient for paracellular movement of glucose from the blood/interstitium to the ASL. Glucose uptake via glucose transporters (GLUTs) is maintained by metabolism, which generates low intracellular glucose. We propose that this occurs predominantly by HKII-driven conversion of glucose to glucose-6-phosphate (G-6-P) and glycolysis. When blood glucose levels are raised to 15 mM (hyperglycemia), there is increased paracellular movement of glucose into the ASL. Increased glucose uptake elevates HKII activity at the mitochondria, increasing G-6-P, glycolysis, and glycogen synthesis. This effectively reduces intracellular glucose concentration, which maintains a glucose gradient for clearance of glucose from the ASL and prevents transcellular efflux into the ASL. Inhibition of HKII with BrPy elevates intracellular glucose, but concentrations remain low compared with external glucose concentration indicating additional contribution of HKI/III and the HK-independent polyol pathway to glucose metabolism. MCTs, monocarboxylate lactate-H^+^ cotransporters; TCA, tricarboxylic acid cycle.

Glucose concentration in washes from the ASL of cell cultures grown at air-liquid interface was increased from 3.6 ± 0.7 to 45.2 ± 1.7 μM, *P* < 0.001, *n* = 4 and 7 experiments, respectively) when basolateral glucose was raised from 5 to 15 mM for 6 h. Taking into account the original volume of ASL, these values approximate to 0.5 and 6 mM, respectively, similar to previously published values (12). Treatment with BrPy had no further effect on ASL glucose concentrations. Transepithelial electrical resistance (TEER) was unaffected by treatments (Fig. 8).

## DISCUSSION

Both H441 cells and primary HBECs expressed all three isoforms of hexokinase (HKI, II, and III). This finding was consistent with that described for lung tissue but now further localizes these isoforms to airway epithelial cells (24). HKI is found in most cells and is thought to be the key enzyme driving oxidative phosphorylation and the production of ATP, whereas HKII is thought to be more limited in its expression and associated with insulin-sensitive tissues (9). HKIII is associated with the cytosol and nuclear periphery (32). We found that growth at air-liquid interface or elevation of glucose from 5 to 15 mM had no effect on the observed abundance of any of the individual isoforms consistent with the finding that HKI, II, and III did not change in the lungs of alloxan-induced diabetic rats compared with wild type (24). Furthermore, we did not observe any difference in total cellular abundance of HKII in H441 cells (derived from a papillary adenocarcinoma) compared with HBECs, although it is widely accepted to be upregulated in nonsmall cell lung cancers (23).

HKII is a key enzyme controlling anabolic (glycogen synthesis) and catabolic (glycolysis) pathways in the cell. In muscle cells, it shuttles to the mitochondria in response to elevated extracellular glucose driving glycolysis and glycogen storage (6, 17). The pyruvate mimetic BrPy enters the cell via monocarboxylate lactate-H^+^ cotransporters [present in H441 cells and HBECs (12)] and is a potent inhibitor of glycolysis (7, 8, 34). It is reported to decrease HKII activity by alkylating and dissociating the enzyme from the mitochondrial membrane (7, 8, 34). HKI is also associated with the mitochondrial membrane and is proposed to maintain glycolysis when extracellular glucose levels are low (17). We could find no evidence to support an effect of BrPy on this hexokinase (17). As HKIII is not bound to the mitochondria, BrPy likely has no effect on this isoform. Our finding that BrPy only inhibited 25% of total hexokinase activity (HKI, II, and III) in cell extracts would indicate that it predominantly targeted HKII activity in these cells but that total cellular hexokinase activity includes that of HKI and HKIII. The concentration-effect curve for BrPy also indicated a possibility that BrPy inhibited two hexokinases with differing affinities. The initial inhibition (i.e., that potentially attributable to HKII) had an IC_50_ of ~40 μM. BrPy inhibited glycolysis with a similar IC_50_ of 60 μM. Others have found similar concentrations of BrPy to inhibit glycolysis in other cell types, and this has been attributed to inhibition of HKII (10, 15, 29).

We used the intracellular FRET sensor FLII12Pglu-700µΔ6 because the purified sensor was largely unaffected by pH, had the lowest *K*_d_ (660 mM), and had the highest dynamic range to ascertain whether intracellular glucose could reach levels higher than that detected in ASL (~400 μM; 35). The standard curve we obtained from the sensor expressed in airway cells had a similar *K*_d_. Although we recognize that the measurements of intracellular glucose concentration below 100 μM were toward the limit of detection with this sensor, we found that intracellular glucose concentrations were in the micromolar range in all our cell models. In HBECs grown at air-liquid interface, values were below or equivalent to concentrations we found in the airway surface liquid (~0.4 mM) in vivo and in vitro (3, 13, 37). These findings support our previous proposal that to maintain ASL glucose concentrations at this level, airway epithelial cell intracellular glucose must be similar or lower to drive glucose uptake (11, 13). We did not take the pulsed approach to changing external glucose for FRET analysis, and we found that although there were consistent overall changes in FRET output, we also observed cyclic fluctuations in intracellular glucose that were inhibited by BrPy (18). As generation of glucose-6-phosphate by hexokinases inhibits HKII activity with high affinity (17), we suggest that this phenomenon underpins these changes (28, 36).

Cytochalacin B, which is reported to inhibit glucose transport via GLUT1, 2, 3, and 4, decreased intracellular glucose (2). Inhibition of GLUT1 and 9 by siRNA in hepatocytes had a similar effect (35). We and others previously proposed that glucose uptake in airway cells utilized GLUT1, 2, 4, and 10 (19, 20, 25, 30). As the effect of cytochalasin B on GLUT10 is currently unknown, we suggest that glucose moves into the airway epithelial cell at least via GLUT1/2/4, and rapid metabolism by HKII maintains low intracellular glucose.

A surprising finding of the study was that intracellular glucose decreased with extracellular hyperglycemia. This was associated with an increase in glycolysis (12) consistent with our previous observations, glycogen synthesis, and potentially other glucose utilization pathways such as the polyol pathway. Interestingly, glycogen synthase was stimulated by hyperglycemia in myoblasts but only when glycogen stores were depleted. The calculated glycogen content in our cells was ~10 times lower than that reported for glucose-starved myoblasts. Thus, it is possible that hyperglycemia also stimulates glycogen synthase in airway cells (14). BrPy increased intracellular glucose concentration. As HKII was reported to respond rapidly to changes in external glucose, we propose that HKII is key in directing the fate of glucose in these cells (17). However, intracellular concentration of glucose remained low compared with the external glucose concentration. This, together with the finding that only 25% of cellular hexokinase activity was inhibited by BrPy, indicates roles for hexokinase I and III in maintaining low intracellular glucose concentration in airway cells.

Effective metabolism and low intracellular glucose in airway cells provide a driving force for glucose uptake. We propose that this helps reduce transepithelial glucose concentration gradients and aids clearance of glucose from the ASL via glucose transporters in the basolateral and apical membranes (19, 20). This work focused on short-term changes in extracellular glucose concentration. We have not yet investigated the effect of chronic elevation of glucose (as observed in poorly controlled diabetes) or in lung disease conditions such as cystic fibrosis where glucose metabolism is reportedly compromised (25). Nevertheless, these data support our proposal that during hyperglycemia, glucose predominantly moves across the epithelium into the ASL via the paracellular rather than transcellular route (13, 19).

## GRANTS

This work was funded by a Medical Research Council Collaborative Awards in Science and Engineering (MRC CASE) studentship award with AstraZeneca, Gothenburg, Sweden. J. P. Garnett was funded by a Respiratory Diseases Research Award from the Medical Research Foundation (Grant MRF-091-0001-RG-GARNE).

## DISCLOSURES

No conflicts of interest, financial or otherwise, are declared by the authors.

## AUTHOR CONTRIBUTIONS

D.L.B. conceived and designed research; J.B., J.P.G., V.S., M.G.S.B., and D.L.B. performed experiments; V.S. and M.G.S.B. analyzed data; J.B., J.P.G., and D.L.B. interpreted results of experiments; J.B., J.P.G., V.S., and D.L.B. prepared figures; J.B. and D.L.B. drafted manuscript; D.L.B. edited and revised manuscript; J.B., J.P.G., V.S., M.G.S.B., and D.L.B. approved final version of manuscript.
